# Long Distance Between the Superior Mesenteric Artery Root and Bottom of the External Anal Sphincter Is a Risk Factor for Stoma Outlet Obstruction After Total Proctocolectomy and Ileal‐Pouch Anal Anastomosis for Ulcerative Colitis

**DOI:** 10.1002/ags3.12512

**Published:** 2021-10-13

**Authors:** Ryota Mori, Takayuki Ogino, Yuki Sekido, Tsuyoshi Hata, Hidekazu Takahashi, Norikatsu Miyoshi, Mamoru Uemura, Yuichiro Doki, Hidetoshi Eguchi, Tsunekazu Mizushima

**Affiliations:** ^1^ Department of Gastroenterological Surgery Graduate School of Medicine Osaka University Osaka Japan; ^2^ Department of Therapeutics for Inflammatory Bowel Diseases Graduate School of Medicine Osaka University Osaka Japan; ^3^ Department of Surgery Osaka Police Hospital Osaka Japan

**Keywords:** ileal pouch‐anal anastomosis, small bowel obstruction, stoma outlet obstruction, total proctocolectomy, ulcerative colitis

## Abstract

**Background:**

Stoma outlet obstruction (SOO) is much more common after total proctocolectomy (TPC) and ileal‐pouch anal anastomosis (IPAA) for ulcerative colitis (UC) compared to after rectal surgery for cancer. Few prior reports have evaluated anatomical risk factors for SOO. In this study we aimed to clarify the risk factors for SOO after IPAA, focusing on the anatomical perspective.

**Methods:**

This study included 68 UC patients who underwent IPAA with diverting ileostomy. These cases were analyzed based on clinicopathological factors and computed tomography (CT)‐based anatomical factors.

**Results:**

SOO was identified in 18 patients (26.5%). We compared this SOO group with the non‐SOO group. The two groups significantly differed in sex distribution, and patients in the SOO group tended to have a longer postoperative hospital stay. Regarding surgery‐related factors, patients who underwent two‐stage surgery and experienced high‐output syndrome tended to develop SOO. Analysis of anatomical risk factors revealed that SOO was more common in patients with a longer distance between the root of their superior mesenteric artery and the bottom of the external anal sphincter (rSMA‐bEAS). This tendency remained significant even with adjustment for patient height. In multivariate analyses, adjusted rSMA‐bEAS (>191.0 mm/m) and male sex were independent risk factors associated with SOO.

**Conclusion:**

A long rSMA‐bEAS distance suggests that the mesentery is likely to be under tension. In such cases, surgeons should endeavor to avoid tension in the mesentery as much as possible.

## Graphical abstract

This study included 68 UC patients who underwent IPAA with diverting ileostomy and these cases were analyzed based on clinicopathological factors and CT‐based anatomical factors. Height adjusted the root of their superior mesenteric artery and the bottom of the external anal sphincter (rSMA‐bEAS; >191.0 mm/m) and male sex were independent risk factors associated with SOO. A long rSMA‐bEAS distance suggests that the mesentery is likely to be under tension. In such cases, surgeons should endeavor to avoid tension in the mesentery as much as possible.

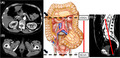

## 1 INTRODUCTION

Ulcerative colitis (UC) is a chronic inflammatory disease of unknown cause, in which the large intestine repeatedly becomes inflamed, and which is affecting an increasing number of patients. The main treatments for UC are medications, such as amino salicylates, steroids, immunosuppressants, and antitumor necrosis factor (TNF)‐α receptor antibody. However, a substantial number of patients require surgery due to toxic megacolon, failing to achieve remission, or tumor formation.^1^

Among patients with UC, the lifetime risk of operation is about 40%,^2^ with the standard surgical procedures including total proctocolectomy (TPC) and ileal‐pouch anal anastomosis (IPAA). Previous reports indicate that IPAA is associated with higher rates of postoperative complications than other types of abdominal surgery, particularly small bowel obstruction (SBO) related to diverting ileostomy.^3^

A diverting ileostomy is created to avoid the risk of anastomotic leakage; however, it sometimes leads to stoma‐associated SBO, termed stoma outlet obstruction (SOO), due to angulations of the ileum near the stoma site. SOO occurs with incidence rates of 20%–40% after IPAA with diverting ileostomy, compared to less than 15% after low anterior resection for rectal cancer.^4^ We reported that the high risk of SOO after IPAA with diverting ileostomy was associated with a short distance from the pouch to the diverting ileostomy, caused by excessive tension of the mesentery.^5^

Based on clinical experience and previous reports, we inferred that taut superior mesenteric vessels after IPAA with diverting ileostomy created a situation in which the ileum could be easily twisted and difficult to restore, due to excessive tension of the mesentery. To date, few studies have evaluated the anatomical risk factors for SOO. Thus, in the present study we aimed to clarify the risk factors for SOO after IPAA with diverting ileostomy, focusing on the anatomical perspective.

## 2 METHODS

### 2.1 Surgical procedure

TPC and hand‐sewn IPAA were performed for UC with cancer or dysplasia, whereas TPC and stapled IPAA were performed for refractory or severe UC. Hand‐sewn anastomosis with mucosectomy was performed in cases with severe inflammation around the anal canal. Some patients initially underwent subtotal colectomy (STC) as an emergency surgery for acute exacerbation, followed by residual rectum resection (RR) and IPAA as a secondary surgery. In accordance with Health and Labor Sciences Research Grants for research on intractable diseases from the Ministry of Health, Labor and Welfare of Japan, the operation could include up to three phases based on whether a diverting ileostomy was created and when closure was performed.

Most procedures involved the creation of a 15‐cm‐long ileal J‐pouch, without dissection of the ileocecal artery (ICA), and a diverting ileostomy on the oral side 30–80 cm side from the IPAA. A diverting ileostomy was created at the umbilicus or right lower abdomen, at the surgeon's discretion. At the site of stoma, a lengthwise incision was made at the rectus muscle fascia, along with a fascial trephination large enough to allow passage of two fingers. Finally, a Turnbull loop ileostomy was fashioned without rotation, in a vertical orientation.

### 2.2 Study population

This study included UC patients who underwent IPAA with a diverting ileostomy at Osaka University Hospital, Japan, between January 2010 and November 2020. Patients with insufficient imaging data were excluded. Informed consent was obtained from all patients before the operation. The study protocol was approved by the Institutional Review Board of Osaka University Hospital (#15028).

### 2.3 CT imaging and measurement of anatomical distances

**FIGURE 1:**
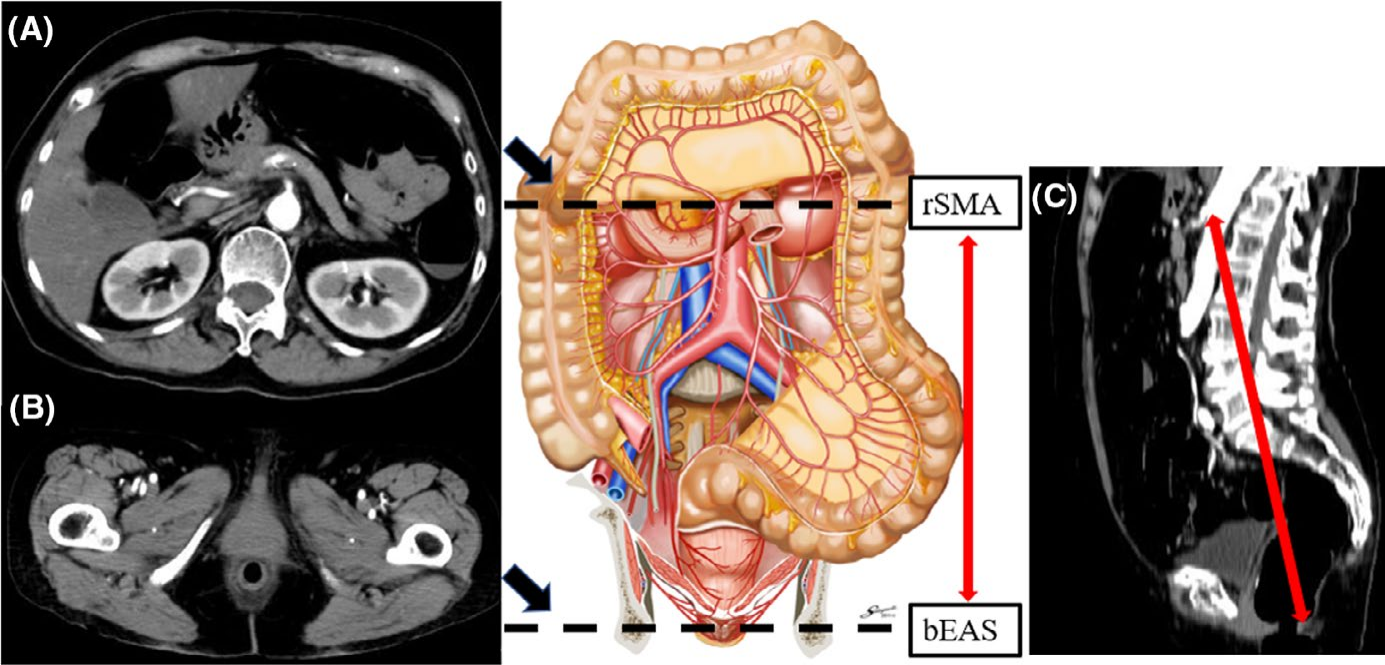
Methods of measuring the distance between the root of the superior mesenteric artery and the bottom of the external anal sphincter (rSMA‐bEAS) on CT images. a: Axial image showing the location of the root of the superior mesenteric artery (rSMA). b: Axial image showing the location of the bottom of the external anal sphincter (bEAS). c: Sagittal image showing the fixed angle such that rSMA and bEAS are on the same slice

**FIGURE 2:**
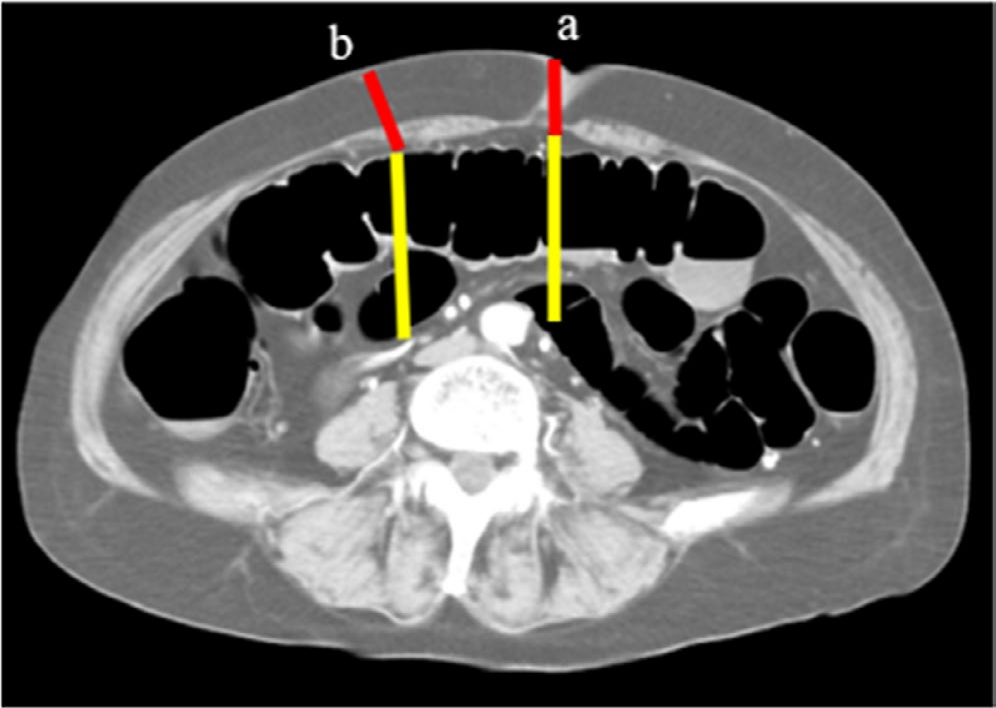
Methods of measuring the abdominal cavity depth and abdominal wall thickness at the site of stoma construction. a: In a case with a diverting ileostomy in the umbilicus. b: In a case with a diverting ileostomy in the lower abdomen

Abdominal and pelvic computed tomography (CT) was performed using 64‐ or 320‐detector row CT. Contrast‐enhanced CT was performed during the portal venous phase (delay, 75 s), after injection of nonionic contrast material (dose, 100 mL of 300 mgI/mL). Axial 3‐ to 5‐mm images were reconstructed and reviewed using a picture archiving and communication system monitor, by two board‐certified gastroenterological surgeons. On the axial image, we identified the location of the root of the superior mesenteric artery (rSMA) and the bottom edge of the external anal sphincter (bEAS; Figure 1a,b). On the sagittal image, we fixed the angle for measurement (Figure 1c). We then measured the distance between the root of their superior mesenteric artery and the bottom of the external anal sphincter (rSMA‐bEAS). For this analysis, rSMA‐bEAS was adjusted according to the patient's height (m) to exclude any influence of height. In the axial image, we also measured the depth of the abdominal cavity, and thickness of the abdominal wall at the stoma site (Figure 2).

### 2.4 Definition of stoma outlet obstruction and high‐output syndrome

**FIGURE 3:**
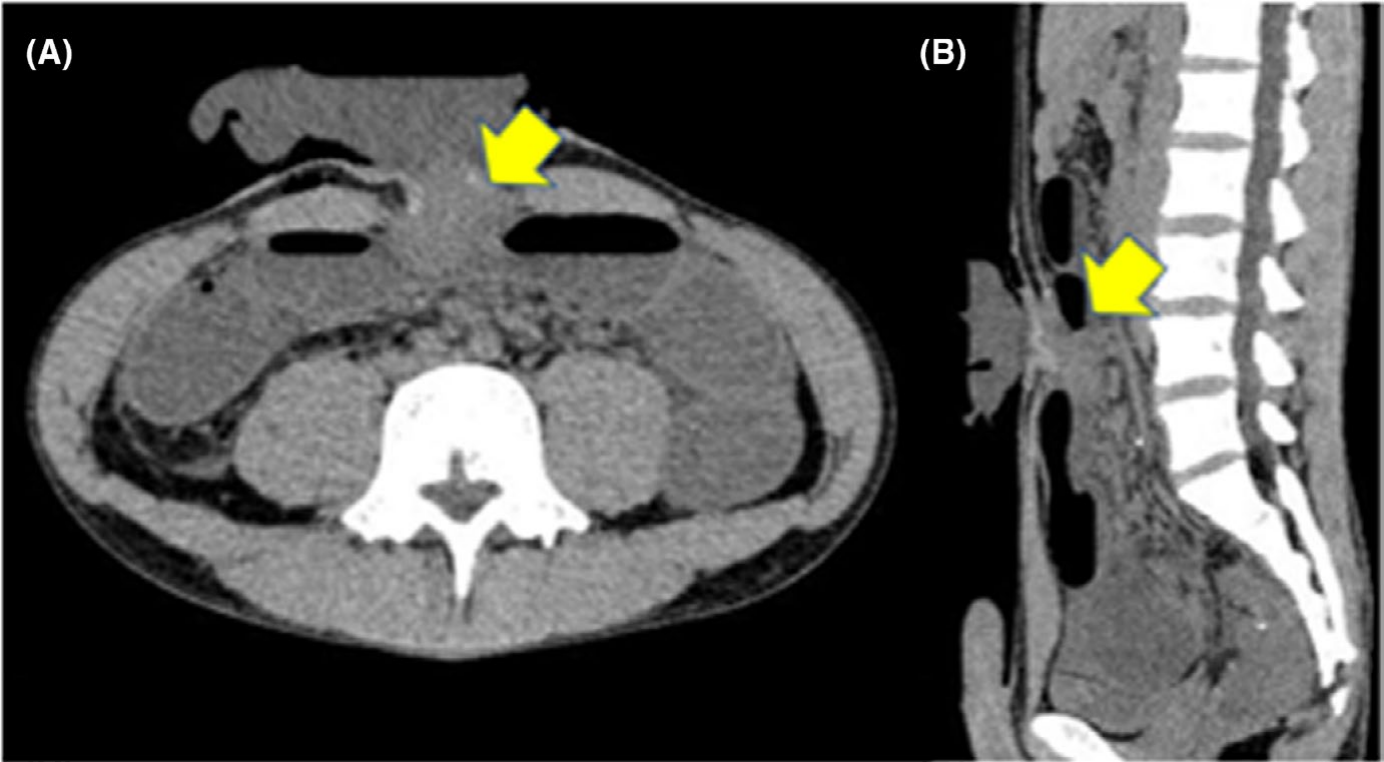
Representative CT images of stoma outlet obstruction (SOO). The arrow points to the area where the intestine was obstructed at the site of a diverting ileostomy. a: Axial image. b: Sagittal image

Stoma outlet obstruction was defined as SBO that showed symptoms of intestinal obstruction, with caliber changes near the ileostomy detected by CT imaging, or recovered by insertion of a tube through the stoma (Figure 3a,b). High‐output syndrome (HOS) was defined as stoma drainage of 2000 mL/d for more than 2 d, or 1500 mL/d for more than 3 d.

### 2.5 Statistical analysis

All statistical analyses were performed using JMP pro 14.0.0 (SAS Institute, Cary, NC). Categorical variables were compared using the chi‐squared test, and continuous variables using the Mann–Whitney *U*‐test and Student's *t*‐test. We assessed the univariate and multivariable associations between factors using binary logistic regression. The cutoff value was determined based on receiver operating characteristic (ROC) curve analysis. *P* < .05 was considered significant.

## 3 RESULTS

### 3.1 Patient characteristics

A total of 78 patients underwent IPAA for UC during the study period. We excluded seven patients without diverting ileostomy, and three patients with incomplete data, such that a total of 68 patients were analyzed in this study. Their clinical characteristics are shown in Table 1. The median age at surgery was 49 y, and mediate disease duration was 9.0 y. The extent of colitis was pancolitis in 57 patients (83.8%), and the main surgical indication was cancer/dysplasia in 27 patients (39.7%).

**TABLE 1:** Clinical characteristics of participants

	n = 68
Sex	
Male	45 (66.2%)
Female	23 (33.8%)
Age at surgery, y	49.0 ± 16.5
Disease duration, y	9 ± 9.7
Body mass index, kg/m^2^	20.8 ± 4.3
Height, m	165.1 ± 9.3
Body weight, kg	57.5 ± 14.2
Smoking	12 (17.6%)
Type 2 diabetes	5 (7.4%)
Extent of colitis	
Pancolitis	57 (83.8%)
Left‐sided colitis	10 (14.7%)
Proctitis	0 (0.0%)
Others	1 (1.5%)
Surgical indication	
Cancer/Dysplasia	27 (39.7%)
Severe/Fulminant	19 (27.9%)
Refractory	22 (32.4%)
Nutritional status (Onodera PNI)	
>40	27 (39.7%)
≦40	41 (60.3%)
Preoperative CRP (mg/dL)	0.5 ± 3.5
Preoperative medication	
Amino salicylate	55 (80.9%)
Anti‐TNFα antibody	26 (38.2%)
Steroid	46 (67.6%)
Immunosuppressant	33 (48.5%)

Note

Data are presented as n (%) or median ± SD.

Abbreviations: CRP, C‐reactive protein; PNI, prognostic nutritional index; TNF, tumor necrosis factor.

Among the 68 patients, SOO was identified in 18 (26.5%). SOO severity was assessed using the Clavien–Dindo classification.^6^ Overall, 12 patients recovered by ileostomy tube drainage with intravenous feeding (Grade II), four patients needed ileus tube insertion (Grade IIIa), and two patients required early stoma closure to manage SOO (Grade IIIb).

We compared the patients in the SOO group (n = 18) with those in the non‐SOO group (n = 50). In terms of patients’ characteristics, sex distribution significantly differed between the two groups (*P* =.003; Table 2). We also assessed surgery‐related and postoperative factors (Table 3). SOO development was significantly associated with undergoing two‐stage surgery (*P* = .01) and experiencing HOS (*P* = .001). The two groups did not differ in terms of operative time (*P* = .23), blood loss (*P* = .86), location of the ileostomy (*P* = .23), or use of laparoscopy (*P* = .95). SOO incidence was not affected by the occurrence of intra‐abdominal abscesses (*P* = .11).

**TABLE 2:** Comparison of clinical characteristics between SOO group and non‐SOO group

	SOO group (n = 18)	Non‐SOO group (n = 50)	*P* value
Sex			.003
Male	17 (94.4%)	28 (56.0%)	
Female	1 (5.6%)	22 (44.0%)	
Age at surgery, y	59.0 ± 21.4	48.5 ± 14.6	.4
Disease duration, y	5.5 ± 7.6	11.0 ± 14.6	.1
Body mass index, kg/m^2^	20.8 ± 3.6	20.8 ± 4.6	.9
Height, cm	169.2 ± 7.2	162.0 ± 9.8	.09
Body weight, kg	60.1 ± 10.8	56.5 ± 15.3	.53
Smoking	3 (16.7%)	9 (18.0%)	.76
Type 2 diabetes	1 (5.6%)	4 (8.0%)	.65
Extent of colitis			.95
Pancolitis	15 (83.3%)	42 (84.0%)	
Left‐sided colitis/Others	3 (16.7%)	8 (16.0%)	
Surgical indication			.43
Cancer/Dysplasia	7 (38.9%)	20 (40.0%)	
Severe/Fulminant	3 (16.7%)	16 (32.0%)	
Refractory	8 (44.4%)	14 (28.0%)	
Nutritional status (Onodera PNI)			.93
>40	7 (38.9%)	20 (40.0%)	
≦40	11 (61.1%)	30 (60.0%)	
Preoperative CRP (mg/dL)	0.5 ± 3.3	0.5 ± 3.5	.91
Preoperative medication			
Amino salicylate	15 (83.3%)	40 (80.0%)	.76
Anti‐TNFα antibody	10 (55.6%)	16 (32.0%)	.08
Steroid	11 (61.1%)	35 (70.0%)	.68
Immunosuppressant	12 (66.7%)	21 (42.0%)	.07

Note

Data are presented as n (%) or median ± SD.

Abbreviations: CRP, C‐reactive protein; PNI, prognostic nutritional index; SOO, stoma outlet obstruction; TNF, tumor necrosis factor.

**TABLE 3:** Comparison of surgery‐related and postoperative factors between SOO group and non‐SOO group

	SOO group (n = 18)	Non‐SOO group (n = 50)	*P* value
*Surgery‐related factors*			
Operation time, min	385.5 ± 158.7	368.0 ± 179.4	.23
Blood loss (mL)	235.0 ± 979.7	179.4 ± 315.4	.86
Surgical approach			.95
Open	1 (5.6%)	3 (6.0%)	
Laparoscopy	17 (94.4%)	47 (94.0%)	
Anastomosis (IPAA)			.69
Hand‐sewn	8 (44.4%)	25 (50.0%)	
Stapled	10 (55.6%)	25 (50.0%)	
Strategy			.01
2‐stage surgery (TPC)	17 (94.4%)	32 (64.0%)	
3‐stage surgery (STC + RR)	1 (5.6%)	18 (36.0%)	
Site of stoma construction			.23
Umbilicus	13 (72.2%)	28 (56.0%)	
Right lower abdomen	5 (27.8%)	22 (44.0%)	
*Postoperative factors*			
Intra‐abdominal abscess	2 (11.1%)	1 (2.0%)	.11
High‐output syndrome^a^	14 (77.8%)	17 (34.0%)	.002

Note

Data are presented as n (%) or median ± SD.

IPAA, ileal pouch‐anal anastomosis; RR, residual rectal resection; SOO, stoma outlet obstruction; STC, subtotal proctocolectomy; TPC, total proctocolectomy.

^a^High‐output syndrome: stoma drainage of 2000 mL/d for more than 2 d or 1500 mL/d for more than 3 d.

In our analysis of anatomical risk factors, we found that patients with a longer rSMA‐bEAS distance were more likely to develop SOO (*P* = .001; Table 4). This tendency remained significant even with adjustment for patient height (*P* = .002). SOO was not significantly correlated with abdominal wall thickness (*P* = .28) or abdominal cavity depth at the site of stoma (*P* = .73).

**TABLE 4:** Comparison of anatomical factors between SOO group and non‐SOO group

	SOO group (n = 18)	Non‐SOO group (n = 50)	*P* value
Thickness of abdominal wall (mm)	18.3 ± 11.7	23.4 ± 9.2	.28
Depth of the abdominal cavity (mm)	66.0 ± 17.8	62.2 ± 24.1	.73
rSMA‐bEAS (mm)	322.2 ± 22.0	294.0 ± 22.1	.001
Height adjusted rSMA‐bEAS (mm/m)	191.8 ± 9.0	180.5 ± 10.7	.002

Note

Data are presented as median ± SD.

Abbreviations: bEAS, bottom of the external anal sphincter; rSMA, the root of superior mesenteric artery; SOO, stoma outlet obstruction.

We performed univariate and multivariate analyses using four risk factors: sex, surgical strategy, HOS, and rSMA‐bEAS (Table 5). The results showed that SOO was independently associated with adjusted rSMA‐bEAS (>191.0 mm/m; *P* = .004; odds ratio [OR]: 4.5; 95% confidence interval [CI]: 2.3–73.7) and male sex (*P* = .01; OR: 25.9; 95% CI: 2.1–318.5).

**TABLE 5:** Univariate and multivariate association with SOO

	SOO group (n = 18)	Non‐SOO group (n = 50)	Univariate analysis		Multivariate analysis	
			*P* value	Odds ratio (95% CI)	*P* value	Odds ratio (95% CI)
Sex			0.003	13.4	0.01	25.9
Male	17 (94.4%)	28 (56.0%)		(1.6–108.3)		(2.1–318.5)
Female	1 (5.6%)	22 (44.0%)				
Strategy			0.01	9.6	0.09	9.1
2‐stage procedure (TPC)	17 (94.4%)	32 (64.0%)		(1.2–77.9)		(0.7–114.8)
3‐stage procedure (STC + RR)	1 (5.6%)	18 (36.0%)				
High‐output syndrome			0.002	6.8	0.17	2.9
Yes	14 (77.8%)	17 (34.0%)		(1.9–23.9)		(0.6–13.5)
No	4 (22.2%)	33 (66.0%)				
Adjusted rSMA‐bEAS, mm/m			0.001	6.3	0.004	13.1
>191.0	11 (61.1%)	10 (20.0%)		(1.9–20.3)		(2.3–73.7)
≤191.0	7 (38.9%)	40 (80.0%)				

Note

Data are presented as n (%).

bEAS, bottom of the external anal sphincter; RR, residual rectal resection; rSMA, root of superior mesenteric artery; SOO, stoma outlet obstruction; STC, subtotal proctocolectomy; TPC, total proctocolectomy.

## 4 DISCUSSION

UC is an inflammatory bowel disease that has shown a rising incidence and prevalence in industrialized countries over recent years.

The standard surgical procedure for UC is TPC. TPC procedures can be divided into three categories depending on the included stages. One‐stage surgery is IPAA without ileostomy. Two‐stage surgery includes TPC with ileostomy + stoma closure, or STC with ileostomy + RR without ileostomy. Three‐stage surgery includes STC with ileostomy + RR with ileostomy + stoma closure. In a large‐scale study, it was reported that about 75% of patients have a diverting ileostomy, and that one‐stage surgery is not common.^5^ In our hospital, about 90% of patients underwent creation of a diverting ileostomy. Overall, of the 68 patients in our study, 18 (26.5%) experienced SOO.

The most common reconstruction approach is IPAA using an ileal J‐pouch.^7^ IPAA can involve shortening of the intestinal tract, with correspondingly increased tension on the mesentery. The major difficulty in IPAA arises when the pouch does not quite reach the anal canal for anastomosis.^7^ Some reports have demonstrated methods to resolve this situation, including sufficient mobilization of the small intestine, division of the serosa of the mesentery, and division of blood vessels to stretch the mesentery.^7^, ^8^ It has previously been reported that the distances from the rSMA to the terminal of the ICA (tICA), and from the tICA to the anal verge are associated with anastomosis difficulty.^7^ These findings suggest that SMA could be an indicator of mesenteric length.

Previously reported risk factors for SBO (including SOO) after TPC have included laparoscopic surgery, loop ileostomy, low BMI, rectus abdominus wall thickness at the stoma‐penetrating site, HOS, and the distance from the ileal pouch inlet to the loop‐ileostomy (<30 cm).^4^, ^5^, ^9^, ^10^, ^11^ The higher incidence of SBO after laparoscopic surgery for UC is in contrast with the lower incidence of SBO after laparoscopic surgery for colorectal cancer compared with open surgery.^12^ This discrepancy is caused by the fact that the small intestine can more easily twist due to the increased movable space after TPC and decreased postoperative adhesions from laparoscopic surgery.^9^, ^13^ In our present study, very few patients underwent open surgery (5.9%), and thus there was no significant difference between the two groups.

Comparing clinical characteristics between the SOO group and the non‐SOO group revealed that male sex, two‐stage surgery, and experienced HOS were more common in the SOO group. Anatomical analysis showed that SOO development was more common in patients with a longer rSMA‐bEAS distance. This tendency remained significant even with adjustment for patient height. Univariate and multivariable analysis revealed that SOO was independently associated with adjusted rSMA‐bEAS (>191.0 mm/m) and male sex. Together with previous findings, our present results suggest that SMA is the defining factor of mesenteric length, and that mesenteric tension not only makes anastomosis more difficult but also increases the risk of developing SOO. After IPAA with diverting ileostomy, fixation of the mesentery with excessive tension and free movement of the small intestine will lead to a twisted ileum and make it difficult to restore the original position. The fact that women are less likely to suffer from postoperative SOO may be due to the fact that they are more prone to visceral ptosis.^14^ This is the first study to identify a risk factor for SOO from the anatomical perspective.

Our study has several limitations. First, these results were based on a single‐center retrospective study with a small sample size. Second, we estimated mesenteric tension based only on measurement of the rSMA‐bEAS distance. Accurate estimation of the intestinal membrane tension would require measurement of the length of the blood vessels via 3D reconstruction. However, the aim of our present study was to predict the risk of SOO before surgery using simple imaging; therefore, we did not measure blood vessel lengths in this study.

In conclusion, in this study we demonstrated that male sex and longer rSMA‐bEAS distance (indicating greater mesentery tension) were risk factors for SOO after IPAA with diverting ileostomy for UC. These findings suggest that surgeons should endeavor to reduce tension in the mesentery by using an applicable technique as much as possible.

## DISCLOSURE

Funding information: This work was supported by a research grant from the Osaka Medical Research Foundation for Intractable Disease.

Conflict of Interest: The authors declare no conflicts of interest for this study.

Approval of the Research Protocol: The study protocol was approved by the Institutional Review Board of Osaka University Hospital.

Informed Consent: Informed consent was obtained from all patients before the operation.

Registry and the Registration: No. of the study/Trial: #15028.

Author contributions: R.M. and T.T. collected the data; R.M. drafted the article; T.O. and T.M. helped finalize the article. Y.S., T.H., H.T., N.M., M.U., Y.D., H.E., and T.M. proofread the content; and T.M. gave final approval of the article. All authors have read and approved the final article.

## References

[1] Uchino M , Ikeuchi H , Hata K , Okada S , Ishihara S , Morimoto K , et al. Changes in the rate of and trends in colectomy for ulcerative colitis during the era of biologics and calcineurin inhibitors based on a Japanese nationwide cohort study. Surg Today. 2019;49(12):1066–73. 31309329 10.1007/s00595-019-01845-2

[2] Yang Z , Wu Q , Wu K , Fan D . Meta‐analysis: pre‐operative infliximab treatment and short‐term post‐operative complications in patients with ulcerative colitis. Aliment Pharmacol Ther. 2010;31(4):486–92. 19925496 10.1111/j.1365-2036.2009.04204.x

[3] Truelove SC , Witts LJ . Cortisone in ulcerative colitis; final report on a therapeutic trial. Br Med J. 1955;2(4947):1041–8. 13260656 10.1136/bmj.2.4947.1041 PMC1981500

[4] Okada S , Hata K , Emoto S , Murono K , Kaneko M , Sasaki K , et al. Elevated risk of stoma outlet obstruction following colorectal surgery in patients undergoing ileal pouch‐anal anastomosis: a retrospective cohort study. Surg Today. 2018;48(12):1060–7. 30046881 10.1007/s00595-018-1698-8

[5] Mizushima T , Kameyama H , Watanabe K , Kurachi K , Fukushima K , Nezu R , et al. Risk factors of small bowel obstruction following total proctocolectomy and ileal pouch anal anastomosis with diverting loop‐ileostomy for ulcerative colitis. Ann Gastroenterol Surg. 2017;1(2):122–8. 29863130 10.1002/ags3.12017 PMC5881312

[6] Clavien PA , Sanabria JR , Strasberg SM . Proposed classification of complications of surgery with examples of utility in cholecystectomy. Surgery. 1992;111(5):518–26. 1598671

[7] Ohira G , Miyauchi H , Narushima K , Kagaya A , Mutou Y , Saitou H , et al. Predicting difficulty in extending the ileal pouch to the anus in restorative proctocolectomy: investigation of a simple predictive method using computed tomography. Colorectal Dis. 2017;19(1):O34–8. 27943576 10.1111/codi.13575

[8] Uraiqat AA , Byrne CM , Phillips RK . Gaining length in ileal‐anal pouch reconstruction: a review. Colorectal Dis. 2007;9(7):657–61. 17824985 10.1111/j.1463-1318.2006.01181.x

[9] Ohira G , Miyauchi H , Hayano K , Kagaya A , Imanishi S , Tochigi T , et al. Incidence and risk factor of outlet obstruction after construction of ileostomy. J Anus Rectum Colon. 2018;2(1):25–30. 31583319 10.23922/jarc.2017-034 PMC6768823

[10] Hara Y , Miura T , Sakamoto Y , Morohashi H , Nagase H , Hakamada K . Organ/space infection is a common cause of high output stoma and outlet obstruction in diverting ileostomy. BMC Surg. 2020;20(1):83. 32345295 10.1186/s12893-020-00734-7 PMC7189461

[11] Takeda M , Takahashi H , Haraguchi N , Miyoshi N , Hata T , Yamamoto H , et al. Factors predictive of high‐output ileostomy: a retrospective single‐center comparative study. Surg Today. 2019;49(6):482–7. 30594951 10.1007/s00595-018-1756-2 PMC6526144

[12] Matsuda T , Endo H , Inomata M , Hasegawa H , Kumamaru H , Miyata H , et al. Clinical outcome of laparoscopic vs open right hemicolectomy for colon cancer: a propensity score matching analysis of the Japanese National Clinical Database. Ann Gastroenterol Surg. 2020;4(6):693–700. 33319160 10.1002/ags3.12381 PMC7726676

[13] Dolejs S , Kennedy G , Heise CP . Small bowel obstruction following restorative proctocolectomy: affected by a laparoscopic approach? J Surg Res. 2011;170(2):202–8. 21474147 10.1016/j.jss.2011.03.004 PMC3326606

[14] Hardisty RH . The management of visceroptosis. Can Med Assoc J. 1925;15(2):158–63. 20315279 PMC1708066

